# Effects of pulmonary rehabilitation on cardiac magnetic resonance parameters in patients with persistent dyspnea following pulmonary embolism

**DOI:** 10.1016/j.ijcha.2022.100995

**Published:** 2022-03-23

**Authors:** J. Gleditsch, Ø. Jervan, S. Haukeland-Parker, M. Tavoly, O. Geier, R. Holst, F.A. Klok, H.H. Johannessen, W. Ghanima, E. Hopp

**Affiliations:** a Department of Radiology, Østfold Hospital, Kalnes, Norway; b Department of Cardiology, Østfold Hospital, Kalnes, Norway; c Department of Physical Medicine and Rehabilitation, Østfold Hospital, Kalnes, Norway; d Institute of Clinical Medicine, University of Oslo, Oslo, Norway; e Department of Medicine, Sahlgrenska University Hospital, Gothenburg, Sweden; f Division of Radiology and Nuclear Medicine, Oslo University Hospital, Oslo, Norway; g Department of Medicine – Thrombosis and Hemostasis, Leiden University Medical Center, Leiden, the Netherlands; h Østfold University College, Fredrikstad, Norway; i Internal Medicine Clinic, Østfold Hospital, Kalnes, Norway; j Department of Hematology, Oslo University Hospital and Institute of Clinical Medicine, University of Oslo, Oslo, Norway

**Keywords:** Cardiac Magnetic Resonance, Post pulmonary embolism syndrome, Dyspnea, Pulmonary rehabilitation, Heart

## Abstract

**Background:**

Persistent dyspnea and reduced exercise capacity is common in pulmonary embolism (PE) survivors. Although improved right ventricular function after pulmonary rehabilitation has been demonstrated in chronic thromboembolic pulmonary hypertension, it is still unknown whether a similar effect also occurs in other patients with dyspnea after pulmonary embolism.

**Purpose:**

The aim of this study was to explore potential effects of a pulmonary rehabilitation program on cardiac structure and function as assessed with cardiac magnetic resonance (CMR).

**Material and methods:**

Twenty-six PE survivors with persistent dyspnea were included. Right and left ventricular assessment with CMR was performed before and after an eight-week pulmonary rehabilitation program.

**Results:**

Dyspnea as measured by the Shortness of Breath Questionnaire improved significantly after rehabilitation: 15 (IQR: 7–31) versus 8 (IQR: 3–17). Absolute right ventricular global longitudinal strain by CMR was reduced from 19% to 18% (95% CI of difference: 0–3 percent points), and absolute RV lateral strain from 26% to 24% (95% CI of difference: 1–4 percent points). Right ventricular mass was reduced after rehabilitation from 49 g to 44 g (95% CI of difference: 2–8 g).

**Conclusion:**

Although there was a substantial improvement in dyspnea after rehabilitation, we found only a minor reduction in absolute right ventricular longitudinal strain and right ventricular mass. No other CMR parameter changed. We therefore suggest that rehabilitation effect of in this patient group was not primarily mediated by cardiac adaptions.

## 1 Introduction

Recent studies have revealed that up to 50% of pulmonary embolism (PE) survivors suffer from varying degrees of persistent dyspnea, reduced exercise capacity and reduced Health Related Quality of Life (HRQoL) [1], [2], [3], [4], [5]. The term Post Pulmonary Embolism Syndrome (PPES) has been proposed to collectively describe the spectrum of sequelae after PE that ranges from CTEPH to unexplained persistent dyspnea and functional impairment [1], [6]. However, the focus of research in follow-up studies of patients with PE has been chronic thromboembolic pulmonary hypertension (CTEPH), which affects only 3–4% of PE survivors [7]. Apart from CTEPH, the exact causes and underlying pathophysiology of persistent dyspnea in patients with a history of PE have not yet been properly explored. Some studies have indicated that post-PE cardiopulmonary abnormalities may explain development of persistent dyspnea in PE patients [8], [9], whereas other studies have suggested general deconditioning to be the main explanatory factor for persistent dyspnea following PE [10], [11].

Previous studies have indicated that persistent dyspnea in PPES is associated with reduced exercise capacity, suggesting a possible receptivity for an intervention such as cardiopulmonary rehabilitation [2]. Pulmonary rehabilitation has demonstrated a reduction in respiratory symptoms and improved physical function in pulmonary hypertension as well as in other patient groups including chronic obstructive pulmonary disease (COPD), lung cancer, and cystic fibrosis [12], [13], [14], [15]. Furthermore, improvement of echocardiographic global right ventricular function after training has been demonstrated in patients with heart failure and COPD [16], [17]. In one study, cardiac index improved after a 12-week pulmonary rehabilitation program, but there was no change in echocardiographic parameters [18]. In rats with experimentally induced pulmonary hypertension, improved right ventricular function after exercise training has been demonstrated [19].

Cardiac Magnetic Resonance (CMR) provides an insight to structural alterations of the cardiac anatomy with high accuracy and is a volumetric method that facilitates precise volume calculations [20], [21]. CMR is not limited by the acoustic window, which means that CMR gives a complete overview of the heart regardless of the patient's constitution. CMR image acquisition is standardized, and the opportunities for post-processing are excellent. The role and usefulness of CMR has previously been studied in the diagnostic work-up of pulmonary arterial hypertension [22], [23].

The current patient group is still puzzling, with unanswered questions on mechanism behind symptoms and possible treatment. Although small studies have suggested a benefit of exercise training on patients with dyspnea following PE [24], [25], the physiologic mechanisms related to this effect is not known. Therefore, we consider CMR suitable for the work-up of this patient group, providing precise measurement and potentially revealing subtle change of cardiac function during an exercise period, irrespective whether the basic measurements were within pathological range or not.

The aim of this study was to quantify any cardiac alteration after pulmonary rehabilitation in people with dyspnea following PE, as assessed with CMR. Our hypothesis was that cardiac function improved after rehabilitation, as measured by right and left ventricular myocardial strain.

## 2 Material and methods

### 2.1 Study design

This is a sub-study of the ongoing PeRehab study (clinicaltrials.gov - NCT03405480) [26]. In this sub-study, we examined a cohort of patients with persistent dyspnea after PE with CMR before and after an 8-week pulmonary rehabilitation program.

The project was approved by the Regional Committee for Medical and Health Research Ethics in Norway (REK 2017/1940) and all participants signed an informed written consent form prior to inclusion.

### 2.2 Study population

Patients were identified from the Østfold Thrombosis Registry (TROLL), which has included all venous thromboembolism (VTE) patients diagnosed at Østfold Hospital in Norway since 2005.

The inclusion criteria were as follows: (1) age 18–75 years; (2) diagnosed acute PE (greater than isolated sub-segmental) by computed tomography pulmonary angiography (CTPA); (3) 6–72 months since diagnosis of PE; (4) persistent dyspnea emerged after the PE episode (Table 1).

After acquiring informed consent, patients were subjected to clinical evaluation (interviews and a self-completed questionnaire) to determine the presence or absence of dyspnea. If dyspnea was confirmed, the extent of dyspnea was graded using the modified Medical Research Council (mMRC) dyspnea scale which uses five statements to describe the patient’s respiratory disability (ranging from 0 to 4, where 4 represents the worst possible state) [27]. Only patients reporting new onset or worsened dyspnea after the acute PE episode and mMRC dyspnea score ≥ 1 were included in the present sub-study.

Exclusion criteria included CTEPH, severe cardiac and pulmonary disease, heart failure with reduced or preserved ejection fraction (EF) according to European Society of Cardiology (ESC) guidelines [28], shortened life expectancy and contraindications to CMR or contrast medium. Table 1 displays a complete list of exclusion criteria.

**Table 1:** Inclusion and exclusion criteria.

**Inclusion criteria**
• Age 18–75 years • Objectively diagnosed symptomatic PE (greater than isolated sub-segmental PE) by CTPA 6 months to 6 years before inclusion • Self-reported dyspnea that occurred or worsened after the acute PE episode that had persisted for at least 6 months, and with mMRC dyspnea scale score ≥ 1 • Signed informed consent
**Exclusion criteria**
• Patients with known pulmonary diseases including obstructive (COPD GOLD ≥ 2 = FEV1/FVC < 0.7 and FEV1 < 80%) and restrictive pulmonary diseases (total lung capacity < 80%), lung cancer or pleural disease • Heart failure with either reduced EF on primary echocardiography or preserved EF (combination of echocardiographic diastolic heart failure, proBNP > 300 mg/L and symptoms) • Chronic thromboembolic pulmonary hypertension • Significant valvular heart disease • Patients who are unable to perform pulmonary rehabilitation due to old age, physical disability or disease • Patients with history of poor compliance or any condition that would interfere with the ability to comply with the study protocol or to give informed consent e.g. history of drug abuse, excessive alcohol beverage consumption, cognitive dysfunction, or severe psychiatric disease • Active malignancy, i.e. receiving active antimitotic treatment or diagnosed within the past 6 months; or recurrent or metastatic; or inoperable (patients with squamous skin cancer and basal cell carcinoma will not be excluded) • Life expectancy < 3 months • Pregnancy • General contraindications for MRI including non-compatible intracranial vascular clips, cardiac pacemaker or ICD, neurostimulator system, cochlear implant and metallic splinters in the eye • Serious renal failure (GFR < 30 ml/minute) • Previous reactions to MRI contrast agent • Inability to lie in the supine position for 60 min • Significant arrhythmias (such as atrial fibrillation or frequent premature ventricular contractions) • Inability to stop breathing for periods up to 15 s • Body weight > 250 kg or very wide circumference

Patients with established diagnosis of CTEPH were excluded. All eligible patients underwent echocardiography at the first study visit. Patients who had (1) echocardiographic findings consistent with high probability for pulmonary hypertension according to ESC pulmonary hypertension guidelines [29], (2) perfusion defects at ventilation-perfusion (V/Q) scintigraphy, and (3) symptoms consistent with pulmonary hypertension, were referred to right ventricular catheterization (RHT). Patients with findings consistent with CTEPH on RHT were subsequently excluded.

Dyspnea before and after rehabilitation was assessed with the Shortness of Breath Questionnaire (SOBQ) scale [30]. SOBQ is a self-administered rating of dyspnea associated with activities of daily living. Twenty-one items assess the severity of dyspnea during specific activities. Grading of items is determined using a 6-point scale (ranging from 0 = “not at all” to 5 = “maximal or unable to do because of breathlessness”). Total scores range from 0 to 120 with a higher score indicating a higher degree of dyspnea. The minimal clinical important difference (MCID) of the SOBQ is five points [31].

PE clot burden was assessed by Mean Bilateral Proximal Extension of the Clot (MBPEC) based on CTPA images at diagnosis [32]. V/Q scintigraphy was performed according to the European Association of Nuclear Medicine (EANM) guideline [33]. Demographic and clinical data including height, weight, smoking history and comorbidities were recorded at inclusion. Pulmonary Embolism Severity Index (PESI) score was calculated retrospectively using data available from the time of diagnosis [34].

### 2.3 Rehabilitation

Patients performed an outpatient pulmonary rehabilitation program for one hour twice weekly for eight weeks [26]. Experienced physiotherapists developed an individually adapted exercise program for each patient based on individual needs and exercise capacity at baseline, and following training principles of existing pulmonary rehabilitation programs [12]. The exercise component of the rehabilitation program consisted of resistance and endurance training. Interval training ranged from 4 to 8 intervals (lasting 30 s to 4 min) per session. Intervals aimed to be of a moderate to high intensity level, and workload was determined by the patients’ subjective experience of breathlessness as measured to level 6–7 on the Borg dyspnea scale [35]. Patients were advised that they should only be able to speak very short sentences during high intensity, and slightly out of breath, but able to have a conversation when training at moderate intensity. In order to progress and increase training intensity, the endurance training aimed to increase the number of intervals and time spent per interval, as well as reducing resting time between intervals.

Based on previous findings of optimal musculature adaptions in resistance training, the resistance training was performed with high load and few repetitions, i.e. 5 repetition maximum (RM) per set to avoid dyspnea [36], [37], [38]. For the lower extremities, 3–4 sets of each exercise were performed per session focusing on large muscle groups, such as the quadriceps (leg press at 70–95% of 1 RM). For the upper extremities, lower loads and greater number of repetitions and sets were used (65–75% 1 RM, 10–12 repetitions, 2–3 set). Progression included increases in load, number of repetitions and/or sets. In addition, exercises to improve balance, flexibility, breathing exercises and relaxation were provided as required.

### 2.4 Cardiac magnetic resonance

CMR was performed before and after the rehabilitation program on one single Siemens 1.5 T Aera magnet (Siemens Healthcare, Erlangen, Germany) according to established recommendations [21], [22]. Electrocardiograph-triggered steady state free precision cine imaging was acquired during multiple breath-holds in three long axis and 10–12 short axis planes of 6 mm thickness. Thirty phases were retrospectively reconstructed per heartbeat. Late gadolinium enhancement (LGE) images were acquired with a phase sensitive inversion recovery sequence with fixed inversion time in similar image planes as cine imaging. T1-mapping was acquired in the diastolic phase in basal and mid-ventricular short axis planes using the Modified Look Locker Imaging (MOLLI) technique. Post-contrast short-axis T1-mapping was acquired 15 min after the start of intravenous contrast medium injection of 0.15 mmol/kg (0.3 mL/kg) Gadoterate meglumine (Clariscan, GE Healthcare, Chicago, IL, USA or Dotarem, Guerbet, Villepinte, France). Hematocrit blood samples necessary for myocardial extracellular volume (ECV) calculation was obtained immediately before CMR. Detailed imaging parameters are presented in appendix 1.

Endo- and epicardial contours were manually drawn in end-diastole and end-systole on short axis images to measure left and right ventricle (LV and RV) volumes and mass. Contours were refined to minimize the difference between LV and RV stroke volumes. RV mass was measured from end systolic images. LV and RV long axis and short axis mean systolic strain were calculated with feature tracking.

Two myocardial regions of interest were manually drawn on short axis T1 maps to measure mean T1 times: LV septum and lateral wall. T1 time of blood was measured from LV cavity. The reported T1 value of each region was the mean of the measured T1 on the basal and mid-ventricular slice. Hematocrit measurement was obtained immediately before CMR.

All CMR post-processing was performed in Segment version 3.1 (Medviso AB, Lund, Sweden) [39]. CMR measurements and post-processing were performed by a single consultant radiologist with 16 years of CMR experience.

### 2.5 Statistical analysis

Continuous variables were reported by means and standard deviations (SD), or by medians and interquartile ranges (IQR), as appropriate. Paired continuous variables were compared prior and after rehabilitation with Wilcoxon signed-rank test. Proportions were compared with Fisher’s exact test. Confidence intervals (CI) of differences were calculated from 2000 bootstrap replications.

A two-tailed alpha of 5% was considered significant. Missing values were not imputed. All statistical analyses were performed using Stata version 16.1 (StataCorp LLC, College Station, TX, USA).

## 3 Results

Twenty-six patients were recruited between January 1st 2018 and December 31st 2019. All patients completed the planned rehabilitation program.

Table 2 presents demographic and baseline parameters. Median age was 61 years (IQR: 47–68 years) and 62% were male. Mean body mass index (BMI) was 30 ± 5 kg/m^2^. Median time since PE was 2.6 years (IQR: 1.6–4.3 years).

**Table 2:** Demographic and baseline parameters, mean (SD) or median (IQR).

Number of patients, n	26
Age, years, median (IQR)	61 (47–68)
Male gender, n (%)	16 (62%)
Height, cm, mean (SD)	175 (8)
Weight, kg, mean (SD)	90 (16)
BMI, kg/m^2^, mean (SD)	30 (5)
Current smoker, n (%)	0
Previous smoker, n (%)	10 (38%)
Never smoker, n (%)	16 (62%)
Time since PE, years, median (IQR)	2.6 (1.6 – 4.3)
V/Q scintigraphy, positive, n (%)	6 (23%)

**Comorbidity**	
Diabetes, n (%)	0
Coronary arterial disease, n (%)	1 (4%)
Renal insufficiency, n (%)	1 (4%)
Hypertension, n (%)	10 (38%)
Hypothyreosis	3 (12%)

**PE features at diagnosis**	
MBPEC 3–4, n (%)	16 (63%)
Right/Left ventricle ratio, mean (SD)	1.03 (0.27)
PESI-score, median (IQR)	64 (52 – 77)

Abbreviations: SD – Standard deviation; IQR – Interquartile range; V/Q – Ventilation/Perfusion; PE – Pulmonary embolism; BMI – Body Mass Index, MBPEC – Mean Bilateral Proximal Extension of the Clot (MBPEC 3–4 represents the combination of central & bilateral PE), PESI – Pulmonary Embolism Severity Index.

Six patients (23%) had perfusion defects on V/Q scintigraphy, of whom one patient had echocardiographic findings suggestive of pulmonary hypertension. This patient was referred to RHT, upon which CTEPH was excluded (mean pulmonary arterial pressure (mPAP) 20 mmHg, pulmonary capillary wedge pressure (PCWP) 13 mmHg, pulmonary vascular resistance (PVR) 1.1 Wood).

Following rehabilitation, there was a significant reduction in dyspnea as assessed with the SOBQ: Median SOBQ score was 15 (IQR: 7–31) before, versus 8 (IQR: 3–17) after rehabilitation (p = 0.001). There was no significant change in heart rate.

**Fig. 1:**
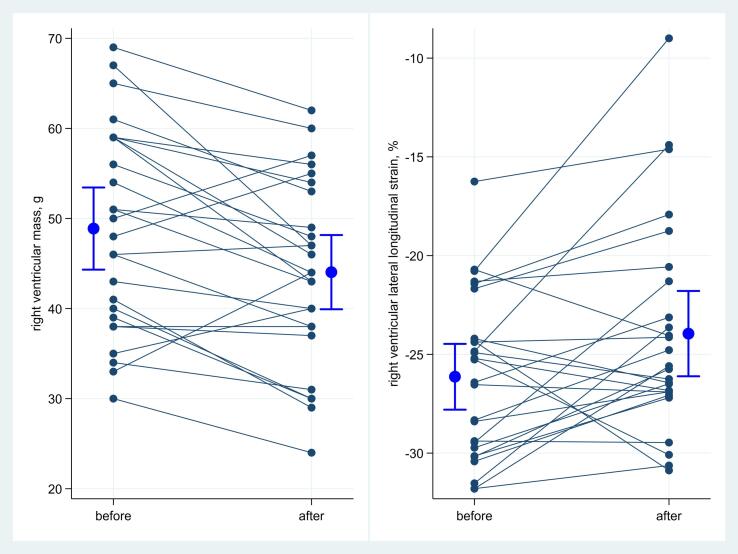
Right ventricular mass (left graph) and right ventricular lateral longitudinal strain (right graph) before and after rehabilitation. Means with 95% confidence intervals (blue). There was a reduction in right ventricular mass and absolute right ventricular lateral strain after rehabilitation (p = 0.01). (For interpretation of the references to colour in this figure legend, the reader is referred to the web version of this article.)

CMR parameters before and after rehabilitation are displayed in Table 3. Absolute RV global longitudinal strain and RV lateral wall longitudinal strain were significantly reduced after rehabilitation by 1 percent point (pp) (95% CI: 0–3 pp) and 2 pp (95% CI: 1–4 pp), respectively. Moreover, there was a significant reduction in RV mass after rehabilitation: 49 g versus 44 g, difference 5 g (95% CI: 2–8 g) (Fig. 1). On stratification by gender, the reduction in RV mass after rehabilitation was only present in men (appendix 2). There was no significant change in stroke volume or cardiac output, LV volumes, EF, strain parameters or LV myocardial mass. There was no change in T1-mapping or ECV metrics. LGE with coronary pattern was found in three patients (12%). Only one of these had a previously diagnosed coronary heart disease and none had symptoms of coronary artery disease (CAD). There was no change in LGE after pulmonary rehabilitation.

**Table 3:** Comparison of cardiac volumes and strain parameters before and after rehabilitation, mean (SD). Differences calculated from 2000 bootstrap replications, mean (95% CI). All p-values calculated with Wilcoxon signed-rank test.

	Before N = 26	After N = 26	Difference (95% CI)	P
Heart rate (s^−1^)	64 (9)	63 (11)	−1 (-4 – 1)	0.21
Stroke volume, ml	77 (20)	79 (18)	2 (-3 – 6)	0.63
Cardiac output, l/min	4.9 (1.1)	4.9 (1.0)	0 (-0.4 – 0.3)	0.82

**Left ventricle**				
End diastolic volume, ml	151 (27)	156 (25)	5 (-1 – 10)	0.20
End systolic volume, ml	74 (16)	77 (14)	3 (-1 – 7)	0.17
Ejection fraction, %	51 (8)	50 (7)	−1 (-3 – 2)	0.78
Left ventricular mass, g	101 (21)	99 (21)	−2 (-6 – 2)	0.61
Global longitudinal strain, %	−16 (3)	−16 (3)	0 (-1 – 1)	0.55
Global circumferential strain, %	−16 (3)	−16 (3)	0 (-1 – 1)	0.75
- Basal	−15 (3)	−16 (3)	0 (-1 – 0)	0.55
- Mid-ventricular	−16 (3)	−15 (4)	0 (-1 – 1)	0.38
- Apical	−18 (4)	−18 (4)	0 (-1 – 2)	0.60

**Right ventricle**				
End diastolic volume, ml	175 (31)	176 (29)	1 (-5 – 7)	0.94
End systolic volume, ml	98 (20)	98 (18)	1 (-4 – 6)	0.58
Ejection fraction, %	44 (8)	44 (7)	0 (-2 – 2)	0.86
Right ventricular mass, g	49 (11)	44 (10)	−5 (-8 – −2)	0.002
Global longitudinal strain, %	−19 (4)	−18 (3)	1 (0 – 3)	0.03
Lateral longitudinal strain, %	−26 (4)	−24 (5)	2 (1 – 4)	0.02
Global short axis strain, %	−10 (4)	−9 (2)	0 (-1 – 2)	0.90
- Basal	−9 (4)	−8 (3)	1 (-1 – 2)	0.92
- Mid-ventricular	−9 (4)	−8 (3)	1 (0 – 2)	0.42
- Apical	−11 (4)	−10 (3)	0 (-2 – 2)	0.96

**T1-mapping**				
Native Septal T1, ms	964 (23)	967 (30)	5 (-6 – 15)	0.65
Native Lateral T1^1^, ms	958 (24)	952 (26)	−3 (-8 – 3)	0.11
ECV Septal^2^, %	22.5 (2.9)	22.4 (3.3)	0.1 (-0.3 – 0.7)	0.99
ECV Lateral^3^, %	21.3 (2.8)	21.6 (2.8)	0.2 (-0.5 – 0.8)	0.37

Abbreviations: ECV, Extra Cellular Volume fraction.

^1^n = 25; ^2^n = 23; ^3^n = 22.

## 4 Discussion

To the best of our knowledge, this is the first study to describe CMR findings after pulmonary rehabilitation in patients with persistent dyspnea after PE. Despite a substantial reduction of patient-reported dyspnea, there were only minimal changes in RV functional parameters. Although statistically significant, the reduction of absolute RV strain was only 1–2 percent points. The difference is within 1SD and in our experience within the margin of uncertainty for strain measurements. We also found a modest reduction in RV mass of 5 g.

In contrast to our findings, an absolute RV global longitudinal strain increase (difference = 3 pp) measured by speckle tracking echocardiography has been demonstrated after four weeks of high intensity exercise training in a group of elderly patients with heart failure with preserved EF [16]. Further, in a study with speckle tracking echocardiography for the evaluation of pulmonary rehabilitation in COPD, RV free wall longitudinal strain improved too (difference = 4.8 pp) [17]. The training intensity in our study was on par with what is common in pulmonary rehabilitation programs [12]. Thus, we believe that that the observed modest change of cardiac function in our study as compared to other studies is not due to differences in training intensity.

Previous studies have demonstrated reduced RV function after high intensity exertion [40], [41], [42], [43]. A *meta*-analysis that included 14 studies and 329 non-dyspneic participants concluded that absolute RV strain was reduced after prolonged exercise while the LV is unaffected [44]. The mechanism may be exercise-induced myocardial fatigue or injury caused by increased pulmonary artery pressure and RV work. Although some studies indicate persistent RV depression, the majority of studies suggest this is reversible. In our study, the patients underwent an individually adapted rehabilitation program based on their extent of dyspnea. This is in contrast to studies that have shown reduced absolute strain where the participants performed physical exertions of much higher intensity, where dyspnea was not a limiting factor. We therefore consider it unlikely that our finding of reduced absolute strain after rehabilitation can be explained by exercise-induced myocardial injury.

We found a reduction in RV mass after rehabilitation. However, it must be emphasized that RV mass is difficult to assess due to the thin and trabecular RV wall. This finding must therefore be interpreted with caution. In a study of 23 untrained men, RV mass increased with 2.7 or 1.4 g after 6 months of endurance or resistance training, respectively [45]. In contrast, a substantial reduction of RV mass was demonstrated after bilateral lung transplant in a group of 10 PAH patients [46]. In line with this, our finding of RV mass reduction might theoretically indicate an adaption to an extracardiac effect of the rehabilitation program. Further, gender differences in RV physiology has been documented [47]. We found it therefore of interest to perform a stratification of our data by gender. This analysis showed that the reduction in RV mass was only statically significant in men. We suggest that these findings might be hypothesis generating warranting further research.

Dyspnea is a non-specific subjective symptom and associated with common diseases such as COPD or CAD. Thus, we considered it important to assess whether undiagnosed/latent COPD or CAD could potentially explain the results. All included patients underwent comprehensive clinical examinations including spirometry and echocardiography prior to inclusion. Patients with diseases associated with dyspnea were excluded including patients with COPD and heart failure. Only three patients were diagnosed with LGE compatible with chronic myocardial infarction after CMR, and only one patient had previously diagnosed (but asymptomatic) coronary artery disease. Of note, our findings of mean left ventricular absolute LV GLS = 16% is below the previous reported normal range (LV: 19%-21% [48]. Absolute RV GLS = 19% is also slightly below the normal range, but for RV GLS the heterogeneity is larger than for LV. These findings indicate that heart failure cannot be completely ruled out in our cohort.

Physical deconditioning is an adaption after a long period with inactivity. However, deconditioning has many facets and is therefore difficult to measure. Although only SOBQ improvement was reported in the present study, it has to be emphasized that the effects of rehabilitation are not limited to improvement of dyspnea or to the increase in exercise capacity. Dyspnea and reduced exercise capacity are considered multifactorial and impaired skeletal muscle function may play a central role in the development of these symptoms rather than cardiopulmonary dysfunction [49], [50]. An augmented “ergoreflex” stimulation due to reduced muscular capacity for aerobic metabolism might result in maladaptive increased ventilation leading to dyspnea and fatigue. We found a major reduction in dyspnea and only modest changes in RV parameters after pulmonary rehabilitation. This indicates that the reduction in dyspnea is not primarily explained by altered cardiac function.

### 4.1 Limitations

First, this was a single site study with a limited number of patients. Second, there was no control arm or reference group; however, our patients acted as their own controls. Third, the effect of rehabilitation as measured by change in SOBQ was prone to confirmation bias. Forth, RHT was only performed in patients with echocardiographic findings consistent with CTEPH. With RHT of all patients with perfusion defects on V/Q scintigraphy, we could have excluded CTEPH with more confidence. This was not accepted for ethical reasons.

Fifth, we cannot rule out that a rehabilitation program of longer duration or with a larger weekly amount of exercise could have resulted in more pronounced changes in CMR parameters.

Finally, the patients were included up to 6 years after the PE event, which may have led to a heterogeneous study population. Dyspnea is common in the general population [51] and is a nonspecific symptom of cardiorespiratory diseases. In some of the participants, dyspnea may have developed independently from their former PE episode. However, we have made efforts to exclude patients with cardiac and pulmonary diseases.

## 5 Conclusion

Despite a substantial, positive effect on dyspnea after pulmonary rehabilitation, we found only a slight reduction in absolute RV longitudinal strain and a modest reduction in RV mass after rehabilitation. Therefore, we suggest that the clinical effect of pulmonary rehabilitation in this patient group was not primarily mediated through cardiac adaptions.

## Declaration of Competing Interest

The authors declare that they have no known competing financial interests or personal relationships that could have appeared to influence the work reported in this paper.

## Figures and Tables

**Table:** 

**Women**	Before n = 10	After n = 10	Difference (95% CI)	p
**Left ventricle**				
End diastolic volume, ml	151 (28)	152 (32)	2 (-5 – 9)	0.77
End systolic volume, ml	71 (16)	71 (18)	0 (-4 – 4)	0.62
Ejection fraction, %	53 (6)	54 (4)	1 (-2 – 4)	0.77
Left ventricular mass, g	84 (11)	85 (14)	1 (-2 – 2)	0.70
Global longitudinal strain, %	−18 (2)	−18 (3)	0 (-2 – 2)	1.00
Global circumferential strain, %	−18 (3)	−18 (3)	0 (-2 – 2)	0.85
**Right ventricle**				
End diastolic volume, ml	169 (33)	172 (1 4 6)	3 (-5 – 11)	0.70
End systolic volume, ml	89 (18)	90 (22)	1 (-4 – 7)	0.56
Ejection fraction, %	47 (5)	48 (3)	1 (-3 – 4)	0.70
Right ventricular mass, g	42 (11)	40 (10)	−2 (-8 – 3)	0.50
Global longitudinal strain, %	−21 (3)	−20 (3)	2 (0 – 4)	0.19
Lateral longitudinal strain, %	−28 (3)	−26 (4)	3 (0 – 5)	0.06
Global short axis strain, %	−9 (2)	−10 (3)	−1 (-2 – 1)	0.38

**Table:** 

**Men**	Before n = 16	After n = 16	Difference (95% CI)	p
**Left ventricle**				
End diastolic volume, ml	151 (27)	157 (21)	7 (-1 – 9)	0.16
End systolic volume, ml	75 (16)	81 (9)	5 (-1 – 11)	0.12
Ejection fraction, %	50 (9)	48 (8)	−1 (-5 – 2)	0.53
Left ventricular mass, g	110 (18)	108 (19)	−1 (-8 – 2)	0.30
Global longitudinal strain, %	−14 (3)	−15 (3)	0 (-2 – 2)	0.43
Global circumferential strain, %	−16 (3)	−15 (3)	0 (-2 – 2)	0.78
**Right ventricle**				
End diastolic volume, ml	179 (30)	179 (24)	0 (-5 – 11)	0.86
End systolic volume, ml	103 (19)	104 (14)	1 (-4 – 7)	0.82
Ejection fraction, %	42 (8)	41 (8)	1 (-3 – 4)	0.94
Right ventricular mass, g	53 (9)	47 (10)	−6 (-9 – −4)	0.0007
Global longitudinal strain, %	−18 (4)	−17 (3)	2 (0 – 3)	0.12
Lateral longitudinal strain, %	−28 (3)	−26 (4)	2 (1 – 4)	0.18
Global short axis strain, %	−10 (4)	−8 (2)	1 (-1 – 3)	0.63

## Appendix 1: Typical imaging parameters

### Cine imaging

Repetition time (TR) = 40 ms (ms), Echo time (TE) = 1.2 ms, field of view (FOV) = 340 × 255 mm, image matrix = 256 × 220, slice thickness = 6 mm, parallel imaging factor (PI) = 2 (GRAPPA), number of excitations (NEX) = 1, partial Fourier transform (PFT) = no, flip angle (FA) = 72°, bandwith = 700 Hz/pixel.

### LGE imaging:

TR = 940 ms, TE = 3.2 ms, inversion time (TI) = 300 ms, FOV = 340x275 mm, image matrix = 256x192, slice thickness = 8 mm, PI = 2 (GRAPPA), NEX = 1, PFT = no, flip angel = 25°, bandwith = 140 Hz/pixel.

### T1-mapping pre contrast with MOLLI technique according to a 5(3)3 scheme:

TR = 293 ms, TE = 1.12 ms, FOV = 360x306 mm, image matrix 256x168, slice thickness = 8 mm, PI = 2 (GRAPPA), NEX = 1, PFT = 7/8, flip angel = 35°, bandwith = 1085 Hz/pixel.

### T1-mapping post contrast with MOLLI technique according to a 4(1)3 scheme:

TR = 373 ms, TE = 1.12 ms, FOV = 360x306 mm, image matrix 256x168, slice thickness = 8 mm, PI = 2 (GRAPPA), NEX = 1, PFT = 7/8, flip angel = 35°, bandwith = 1085 Hz/pixel.

## Appendix 2: CMR parameters stratified by gender

Comparison of cardiac volumes and strain parameters before and after rehabilitation, mean (SD) in **women**. Differences calculated from 2000 bootstrap replications, mean (95% CI). All p-values calculated with Wilcoxon signed-rank test.Comparison of cardiac volumes and strain parameters before and after rehabilitation, mean (SD) in **men**. Differences calculated from 2000 bootstrap replications, mean (95% CI). All p-values calculated with Wilcoxon signed-rank test.
